# Intraoperative real-time display of 3D reconstruction models in robot-assisted partial nephrectomy

**DOI:** 10.1007/s00464-025-12505-7

**Published:** 2026-01-05

**Authors:** Shunsuke Miyamoto, Hiroyuki Shikuma, Ryo Tasaka, Yuki Kohada, Kenshiro Takemoto, Miki Naito, Kohei Kobatake, Yohei Sekino, Hiroyuki Kitano, Kenichiro Ikeda, Keisuke Goto, Akihiro Goriki, Keisuke Hieda, Tetsutaro Hayashi, Nobuyuki Hinata

**Affiliations:** Department of Urology, Graduate School of Biomedical and Health Sciences, Hiroshima University, 1-2-3 Kasumi, Minamiku, Hiroshima, 734-8551 Japan

**Keywords:** 3D reconstruction model, Partial nephrectomy, RAPN, Renal cell carcinoma, Trifecta status

## Abstract

**Objective:**

Robot-assisted partial nephrectomy (RAPN) is a widely used technique for treating renal tumors. However, accurate identification of tumor locations and precise surgical planning remain challenging, particularly in cases with complex anatomy or tumors in difficult-to-reach areas. This study evaluated the usefulness of intraoperative real-time 3D reconstruction models for RAPN.

**Methods:**

We analyzed the data of 57 patients who underwent RAPN using 3D reconstruction models (3D-R group) and 173 patients who underwent RAPN without these models (control group). The models were generated from the preoperative Computed tomography images and displayed on a monitor in the operating room to plan surgical approaches and guide tumor dissection. Propensity score matching was performed to compare the surgical outcomes between the two groups.

**Results:**

The use of real-time 3D reconstruction models significantly improved surgical outcomes in the propensity score-matched cohort. The ischemia time was shorter in the 3D-R RAPN group than in the control group (p = 0.019). Significantly fewer overall and major complications were observed in the 3D-R RAPN group (p = 0.023 and p = 0.041, respectively). Additionally, the 3D-R RAPN group achieved the trifecta status at a significantly higher rate than the control group (p = 0.005).

**Conclusions:**

3D reconstruction models are valuable tools for guiding RAPN, leading to shorter warm ischemia times, fewer complications, and a higher trifecta achievement rate, thereby enhancing the overall quality of care of patients undergoing RAPN.

**Supplementary Information:**

The online version contains supplementary material available at 10.1007/s00464-025-12505-7.

Robot-assisted partial nephrectomy (RAPN) has become increasingly prevalent in the treatment of renal tumors in recent years, with an increasing number of cases of renal cell carcinoma (RCC) [1]. Concurrently, the number of high-complexity cases has increased, making RAPN a highly challenging procedure with a significant risk of complications [2]. Renal tumors exhibit considerable variation in their location, size, and surrounding anatomical structures [3]. Consequently, it is imperative to accurately determine the anatomical position of the tumor and develop a meticulous surgical plan tailored to each individual case. However, accurately identifying the tumor location and devising an appropriate surgical strategy remain challenging, particularly when the anatomical structures are intricate [4].

Preoperative imaging studies, particularly dynamic computed tomography (CT), are crucial for understanding the anatomical structures, tumor location, and their relationships. However, due to the nature of 2D imaging of CT, achieving an accurate understanding of anatomical structures is challenging. Therefore, the surgeon is tasked with converting CT images into three-dimensional (3D) format in their mind. 3D reconstruction models are highly beneficial for overcoming this difficulty [5, 6].

However, 3D reconstructions require close collaboration and support from engineers using specialized software and technology. Facilities where such 3D reconstructions are routinely available in clinical practice remain limited [4, 7, 8].

To overcome this limitation, we collaborated with ZIOSOFT (Tokyo, Japan) to develop a new application for renal surgery using the 3D reconstruction software REVORAS. This software automatically extracts the anatomical structures required for partial nephrectomy and semiautomatically extracts renal tumors. As the majority of 3D reconstruction images are generated automatically, this software requires no specialized skills or engineers, enabling surgeons to independently produce high-precision 3D reconstruction images.

In the present study, we describe how to create a 3D reconstruction model for renal surgeries using the application of REVORAS, compare the surgical outcomes of RAPN using a 3D reconstruction model, and evaluate the usefulness of 3D reconstruction models for renal surgeries.

## Methods

This study was conducted in accordance with the ethical standards of the Declaration of Helsinki and was approved by the Research Ethics Committee of Hiroshima University (authorization number: E2202-0142). Informed consent was obtained using an opt-out method. All procedures received pharmaceutical approval in Japan at the time of study commencement. All surgeons were certified and experienced in robotic surgery using da Vinci or hinotori.

### Patient selection

All of 57 patients who underwent RAPN performed by experienced surgeons using 3D reconstruction models at Hiroshima University Hospital between November 2021 and January 2024, were prospectively enrolled in the 3D-R RAPN group. The 3D-R RAPN group operative outcomes were retrospectively compared with 173 patients who underwent RAPN by experienced surgeons without the aid of real-time 3D reconstruction models between June 2015 and October 2021 as the control RAPN group. To compare the two cohorts as equally as possible, 3D-R and control RAPN patients were matched using propensity score matching (PSM).

### Imaging protocol

All imaging was performed using a 256 × 0.625 mm scanner (Revolution, GE Healthcare, USA) and a 256 × 0.500 mm (Aquilion, Canon, Japan) CT detector. We acquired unenhanced and dynamic CT scans, for corticomedullary, nephrographic, and excretory phases, before RAPN.

### 3D reconstruction models for RAPN

**Fig. 1:**
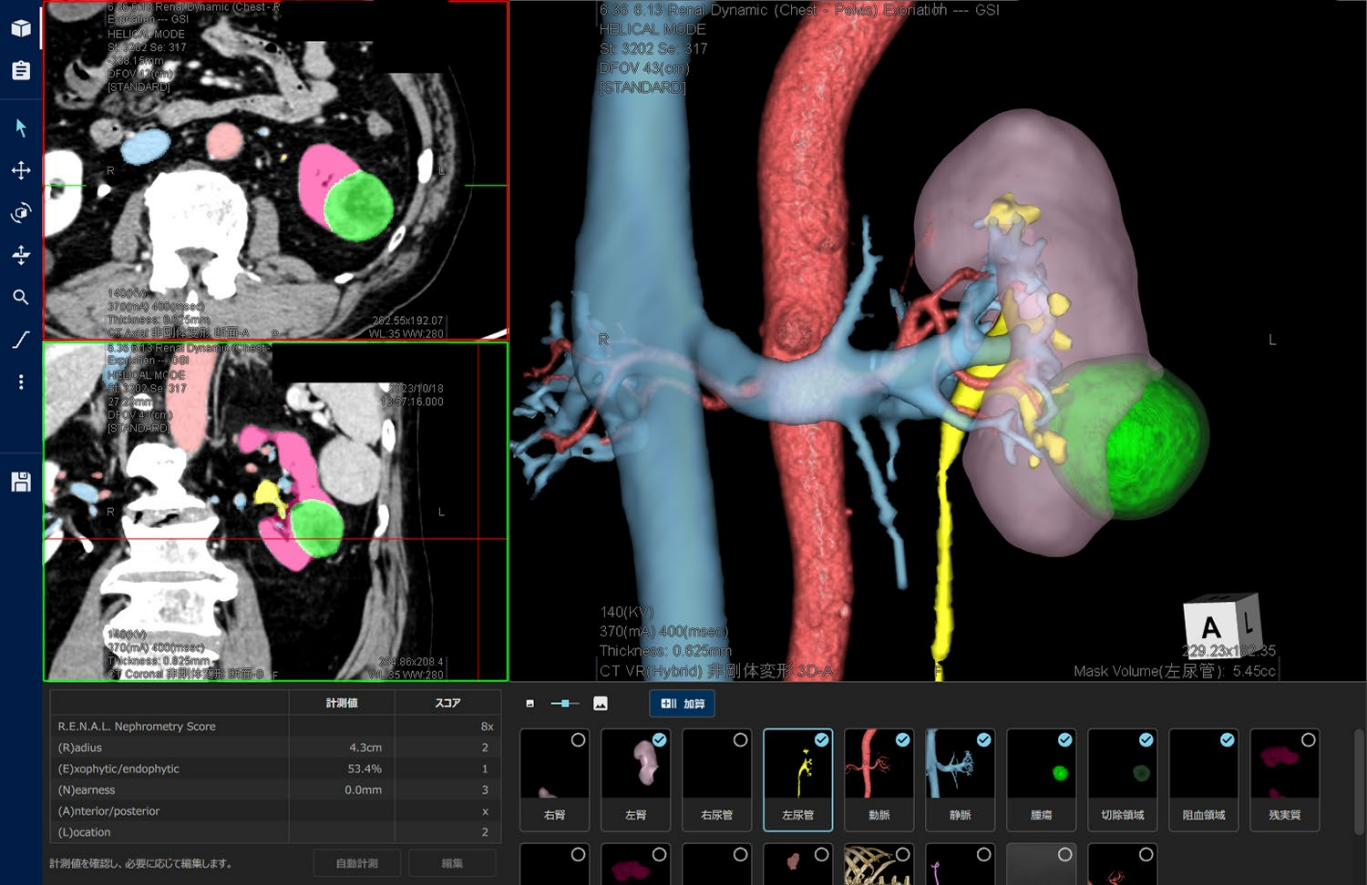
3D reconstruction imaging software. This application renders all the organs required for partial nephrectomy capable of rotation, zooming, and toggling visibility in any direction. It allows for the identification of perfusion areas of renal artery branches, estimation of renal volume after resection, and automatic calculation of the R.E.N.A.L. nephrometry score

The 3D-model reconstruction was performed using REVORAS (ZIOSOFT, Tokyo, Japan) before surgery. REVORAS requires two phases to enhance the CT images of slices smaller than 1.2 mm to build 3D reconstruction models. We made 3D-models using CT images with a thickness of 0.625 mm or 0.5 mm corticomedullary and excretory phases, or nephrographic and excretory phases. In most cases, a 3D reconstruction model can automatically create the anatomical structures necessary for partial nephrectomy, specifically the renal parenchyma, renal arteries, renal veins, urinary tract, aorta, inferior vena cava, gonadal vein, adrenal glands, and other microvessels near the kidneys. Manual adjustments are required in complex cases. 3D reconstruction of renal tumors was performed by manually guiding an area of a sphere around the tumor as assistance, automatically detecting the outline of the tumor, and extracting it according to its shape. The 3D reconstruction models were created by experienced urologists. According to the definition outline, the R.E.N.A.L. nephrometry score was automatically calculated from the 3D model as part of the preoperative information [9]. The 3D reconstruction model allowed the identification of perfusion areas of the renal artery branches and estimation of renal volume after resection. (Fig. 1) Video 1 presents a demonstration of the methodology for generating three-dimensional reconstructed images from a real clinical case, covering the entire process from imaging data acquisition to final model rendering.

**Fig. 2:**
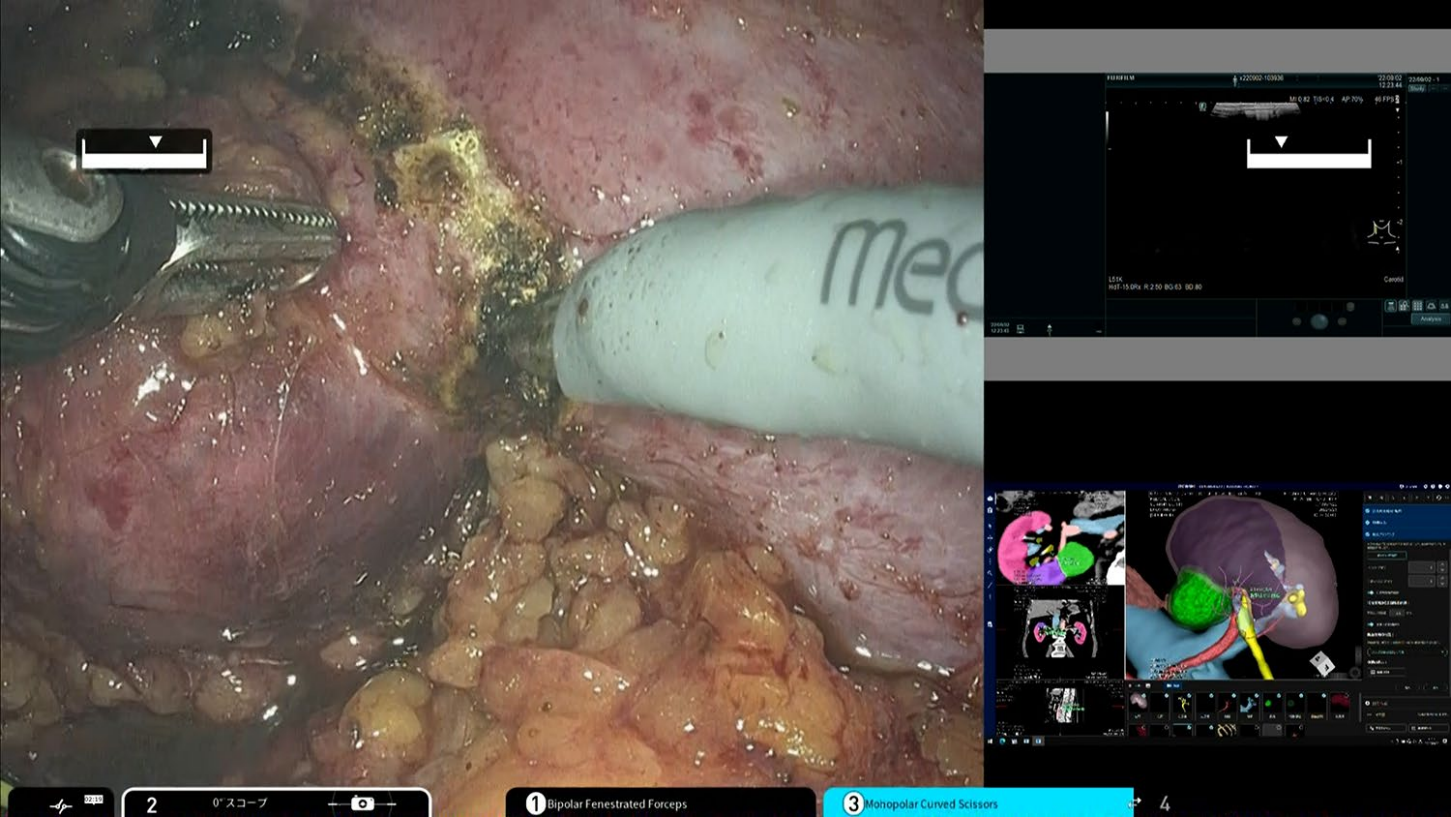
Intraoperative view during robotic surgery. The surgeon could perform the procedure while continuously displaying 3D images using the tiling function

Before surgery, a surgical conference was held where a 3D reconstruction model for each patient was displayed. The 3D model was displayed on a monitor in the operating room during surgery and was tiled to be displayed on the surgical robot. Surgeons can freely rotate and adjust the orientation of the 3D model with the option of toggling the organ labels on or off. The renal parenchyma and veins are semi-transparent. These features facilitate alignment with the intraoperative view, enabling the recognition of anatomical structures that are not directly visible during surgery, thereby providing detailed anatomical insights (Fig. 2).

### Operation

In all cases, five experienced surgeons performed RAPN. Experienced surgeons were defined as those who had performed ≥ 100 RAPN cases, according to the learning-curve analysis described by Bajalia et al. [10]. Surgeries were performed using a surgical robot with hinotori (Medicaroid, Kobe, Japan) and da Vinci Xi/Si (Intuitive Surgical, CA, United States).

In principle, the surgery is performed using an intraperitoneal approach. However, in cases in which adhesions were anticipated due to previous surgeries, a retroperitoneal approach was employed. All the perirenal fat was dissected, leaving only the renal hilum intact. The resection line was determined using ultrasonography with 3D reconstruction models in the selected cases. Either resection or enucleoresection was performed [11, 12]. Vascular control was achieved by clamping the renal arteries. Suturing was performed using barbed sutures with collecting system closure and inner layer renorrhaphy, followed by cortical renorrhaphy using the sliding clip technique.

All pathological evaluations were rigorously performed or validated under the direct supervision of a single board-certified genitourinary pathologist at our institution.

### Preoperative clinical data and perioperative outcomes

All preoperative clinical data and perioperative outcomes were analyzed retrospectively. Preoperative demographics such as age, body mass index, and sex were collected from the medical records of this cohort. Data on tumor characteristics were collected including location, tumor diameter, R.E.N.A.L nephrometry score, preoperative estimated glomerular filtration rate (eGFR), and operative access. perioperative outcomes included operative time (OT), robotic surgery time (RT), warm ischemia time (WIT), estimated blood loss (EBL), intra-operative complications, conversion to radical nephrectomy, and conversion to open surgery. Postoperative renal function was defined as the eGFR measured 6 months after RAPN. Pathological features were also assessed. Data on postoperative complications were collected through a chart review by a medical doctor 90 days after surgery according to the European Association of Urology Guidelines Panel recommendations. Complications were graded according to the Clavien-Dindo system. Renal function was assessed by measuring the eGFR using serum creatinine levels based on the Modification of Diet in Renal Disease equation.

The primary endpoint of this study was the trifecta achievement rate in the propensity score–matched cohort, which was used to evaluate perioperative outcomes in robot-assisted partial nephrectomy (RAPN).

Trifecta was defined as a combination of negative surgical margins, WIT within 25 min, and no perioperative complications [13].

### Statistical analysis

Mann–Whitney U and Fisher’s exact tests were used to evaluate the differences in characteristics and perioperative outcomes between the groups. Primary endpoint was compared using Fisher’s exact test with odds ratios (OR) and 95% confidence intervals (CI).

PSM was performed to eliminate selection biases arising from the non-random assignment of patients to different groups. The covariates for matching were set as factors that could potentially affect postoperative outcomes after nephrectomy: age, body mass index, R.E.N.A.L. nephrometry score, tumor size, and surgical access. The propensity score was derived from a multivariate logistic model that considered the covariates. Based on the resulting propensity score, patients who underwent 3D-R RAPN were matched 1:1 without replacement to control the RAPN group using nearest-neighbor matching within a propensity score-based caliper. A standard caliper size of 0.2 × standard deviation (SD) of logit(propensity score) was used. All statistical analyses were performed using the JMP Pro 14.0.0 (SAS Institute, Cary, NC, USA). Statistical value of p < 0.05 was considered to indicate statistical significance for each comparison.

## Results

**Fig. 3:**
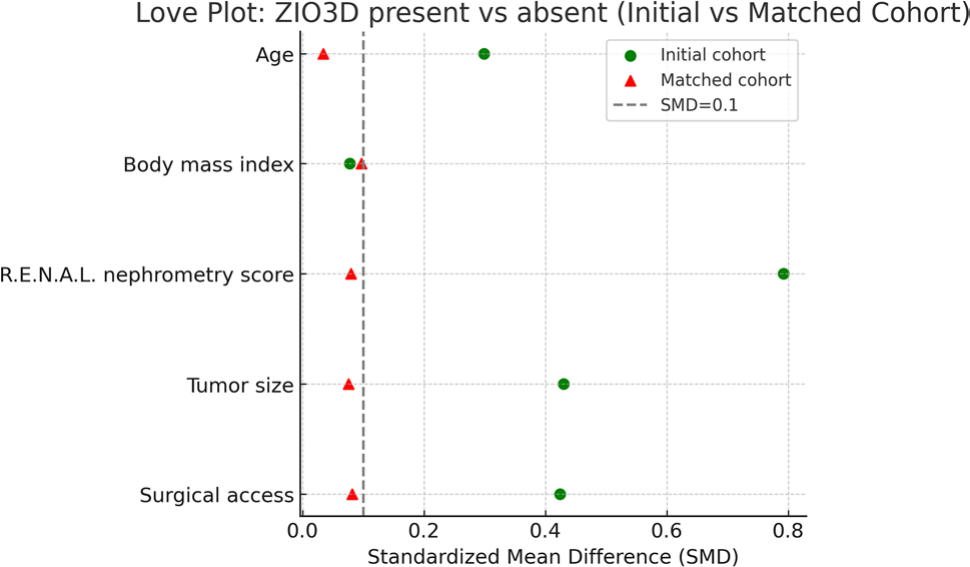
Love plot of covariate balance before and after propensity score matching. Love plot showing standardized mean differences (SMDs) of baseline covariates between control RAPN and 3D-R RAPN before and after propensity score matching. Green circles indicate the initial cohort, and red triangles indicate the matched cohort. The gray dashed line represents an SMD of 0.1

PSM was performed using age, body mass index, R.E.N.A.L. nephrometry score, tumor size, and surgical access as covariates. Matching yielded 50 well-balanced pairs in the 3D‑R RAPN and control groups. All baseline covariates achieved adequate balance after matching, with standardized mean differences < 0.1 across variables (Table 1; Fig. 3).

**Table 1:** Standardized mean differences of baseline covariates before and after propensity score matching

	Standardized mean differences	
Variable	Before	After
Age, years	0.299	0.034
Body mass index	0.078	0.097
R.E.N.A.L. nephrometry score	0.792	0.080
Tumor size, mm	0.430	0.076
Surgical access, Intra vs Retro	0.424	0.082

The demographic and preoperative features of the initial and propensity score-matched cohorts are shown in Table 2. The initial cohort included 173 patients who underwent controlled RAPN and 57 3D-R RAPN. In the initial cohort, patients in the 3D-R RAPN group were older (p = 0.044) and had larger left renal tumors (p = 0.027), larger tumor diameters (p < 0.001), higher R.E.N.A.L nephrometry scores (p < 0.001), lower preoperative renal function (p = 0.005), and higher intraperitoneal access (p = 0.008) than that of the control group.

**Table 2:** Characteristics of patients and tumors between control RAPN and 3D-R RAPN

	Initial cohort			Matched cohort		
	Control RAPN	3D-R RAPN	p	Control RAPN	3D-R RAPN	p
	n = 173	n = 57		n = 50	n = 50	
Patient demographics						
Age, years, median (IQR)	62 (51–70)	64 (57–72)	0.044	62 (54–72)	63 (55.8–71)	0.865
Body mass index,median (IQR)	23.7 (21.4–26.0)	24.20 (21.5–26.6)	0.598	23.69 (21.5–26.2)	23.60 (21.4–27.0)	0.628
Sex, male/female, n (%)	126 (72.8)/47 (27.2)	41 (71.9)/16 (28.1)	0.218	38 (76.0)/12 (24.0)	35 (70.0)/15 (30.0)	0.499
Tumor characteristics						
Location, right/left, n (%)	93 (53.8)/80 (46.2)	21 (36.8)/36 (63.2)	0.027	27 (54.0)/23 (46.0)	19 (38.0)/31 (62.0)	0.418
Tumor diameter, mm, median (IQR)	25 (19.5–33.0)	36 (25.5–45.5)	< 0.001	30 (23–37.25)	35.5 (24.8–44.3)	0.120
RENAL score	7 (4–10)	8 (4–11)	< 0.001	8 (8–9)	8 (7–9.3)	0.689
R 1/2, n (%)	150 (86.7)/23 (13.3)	31(54.4)/26(45.6)		40 (80.0)/10 (20.0)	30 (60.0)/20 (40.0)	
E 1/2/3, n (%)	56 (32.4)/91 (52.6)/26 (15.0)	21 (36.8)/(35.1)/16 (28.1)		10 (20.0)/25 (50.0)/15 (30.0)	19 (38.0)/17 (34.0)/14 (28.0)	
N 1/2/3, n (%)	59 (34.1)/40 (20.1)/74 (42.8)	6 (10.5)/7 (12.3)/44 (77.2)		7 (14.0)/7 (14.0)/36 (72.0)	6 (12.0)/7 (14.0)/37 (74.0)	
A a/p/x, n (%)	74 (40.9)/80 (46.2)/19 (11.0)	20 (35.1)/24 (42.1)/13 (22.8)		23 (46.0)/18 (36.0)/9 (18.0)	18 (36.0)/22 (44.0)/10 (20.0)	
L 1/2/3, n (%)	77 (44.5)/48 (27.8)/48 (27.8)	13 (22.8)/23 (40.4)/21 (36.8)		9 (18.0)/17 (34.0)/24 (48.0)	12 (24.0)/20 (40.0)/18 (36.0)	
4–6/7–9/10–12, n	70/93/10	9/35/13		5/38/7	9/29/12	
Renal function						
Pre operative eGFR, mL/min/1.73m^2^ (IQR)	71.3 (58.3–80.3)	63.2 (57.5–76.0)	0.005	68.5 (55.8–77.3)	64.0 (57.8–76.3)	0.525
Operative access						
Intra/retro, n (%)	83 (48.3)/89 (51.7)	39 (68.4)/18 (31.6)	0.008	30 (60.0)/20 (40.0)	32 (64.0)/18 (36.0)	0.680
Surgical platforms						
da Vinci/hinotori, n (%)	172 (100.0)/0 (0.0)	55 (96.5)/2 (3.5)	0.061	50 (100.0)/0 (0.0)	48 (96.0)/2 (4.0)	0.495

After PSM using the covariates of age, BMI, R.E.N.A.L. nephrometry score, tumor diameter, and surgical access, 50 control and 50 3D-R RAPN patients were included in the cohort. There were no significant differences in demographics or preoperative features between the control and 3D-R RAPN groups in the matched cohort.

Perioperative outcomes of the initial and propensity score–matched cohorts are shown in Table 3. In the initial cohort, the 3D-R RAPN group had a longer operative time (p = 0.020), longer robotic surgery time (p < 0.001), a higher rate of overall complications (p = 0.028), and lower residual renal function (p = 0.036).

**Table 3:** Operative outcomes of patients between control RAPN and 3D-R RAPN

	Initial cohort			Matched cohort		
	Control RAPN	3D-R RAPN	p	Control RAPN	3D-R RAPN	p
	n = 173	n = 57		n = 50	n = 50	
Operative time, min, median (IQR)	207 (178.5–240)	231 (196–262.5)	0.02	228 (201.75–252)	225.5 (193.75–264.75)	0.758
Robot surgery time, min, median (IQR)	119 (90–153.5)	146 (119–173)	< 0.001	137.5 (117.25–161.25)	144.5 (116.75–174.75)	0.580
Warm ischemia time, min, median (IQR)	17 (13–22)	19 (17–21)	0.482	21.5 (17.75–25)	19 (17–21)	0.019
Warm ischemia time < 25 min, n (%)	151 (87.3)	53 (93.0)	0.217	37 (74)	47 (94)	0.006
Estimated blood loss, mL, median (IQR)	62 (30–144.5)	120 (70–231)	0.342	100 (41.25–290.5)	111.5 (60–227.5)	0.404
Surgical margin positive, n (%)	0 (0)	0 (0)	1	0 (0)	0 (0)	1
Complications, n (%)						
Overall	52 (30.1)	9 (15.8)	0.028	18 (36)	8 (16)	0.023
Clavien-Dindo 3	8 (4.6)	0 (0)	0.098	4 (8)	0 (0)	0.041
Clavien-Dindo 4–5	0 (0)	0 (0)	1	0 (0)	0 (0)	1
Transfusion, n (%)	2 (1.2)	0 (0)	0.415	2 (4)	0 (0)	0.094
Conversions to RN or OS n (%)	0 (0)	0 (0)	1	0 (0)	0 (0)	1
Trifecta, n (%)	145 (83.8)	52 (91.2)	0.166	35 (70)	46 (92)	0.005
Postoperative eGFR						
Post/pre eGFR, %, median (IQR)	92.6 (86.6–100)	86.2 (82.2–94.7)	0.036	88.2 (84.8–94.5)	87.3 (82.8–95.3)	0.678

In the matched cohort, the primary endpoint, the trifecta achievement rate, was significantly higher in the 3D-R RAPN group (92% vs. 70%, p = 0.005, OR 4.94 [95% CI 1.55–15.7]). In addition, the 3D-R RAPN group showed a significantly shorter WIT (median 19 vs. 21.5 min, p = 0.019). The mean reduction in WIT was approximately 2.5 min (12%) compared with the control group. The 3D-R RAPN group also had fewer overall complications (16% vs. 36%, p = 0.023) and fewer major complications (0% vs. 8%, p = 0.041). None of the patients required conversion to radical nephrectomy or open surgery.

The pathological and oncological outcomes are summarized in Table 4. There were no significant differences in the main pathology and histological subtypes between the control and 3D-R RAPN groups in either cohort.

**Table 4:** Pathological outcomes between control RAPN and 3D-R RAPN

	Initial cohort			Matched cohort		
	Control RAPN	3D-R RAPN	p	Control RAPN	3D-R RAPN	p
	n = 173	n = 57		n = 50	n = 50	
Main pathology, n (%)						
Benign	12 (6.9)	3 (5.3)	0.650	1 (2)	3 (6)	0.297
Malignant	161 (93.1)	54 (94.7)		49 (98)	47 (94)	
Histologic subtypes, n (%)						
Clear cell	135 (83.9)	47 (87.0)	0.574	40 (81.6)	42 (85.7)	0.284
Non clear cell	26 (16.1)	7 (13.0)		9 (18.4)	5 (10.2)	
Chromophobe	18 (11.2)	4 (7.4)		6 (12.2)	3 (6.1)	
Papillary	8 (5.0)	1 (1.9)		3 (6.1)	0 (0)	
Others	0 (0)	2 (3.7)		0 (0)	2 (4.1)	
pT, n (%)						
1a	139 (80.3)	38 (66.7)		39 (79.6)	34 (69.4)	
1b	16 (9.2)	10 (17.5)		10 (20.4)	8 (16.3)	
2	1 (0.6)	0 (0)		0 (0)	0 (0)	
3	2 (1.2)	6 (10.5)		0 (0)	5 (10.2)	
Positive surgical margins, n (%)	0 (0)	0 (0)		0 (0)	0 (0)	

## Discussion

Since the early 2000s, 3D reconstruction technology has been used in the surgical management of renal cancer [14, 15]. Recent advancements in computational power, processing speed, and cost efficiency have further enhanced the applications of 3D imaging [16]. This technology addresses the inherent limitations of conventional 2D imaging modalities, such as CT, by providing accurate anatomical visualization and overcoming the loss of depth perception [17]. Although numerous studies have reported the benefits of 3D reconstruction in facilitating partial nephrectomy, a definitive consensus on its clinical utility has yet to be established [18].

Porpiglia et al. compared the outcomes of nephron-sparing surgery using intraoperative ultrasound (US) versus 3D reconstructed imaging. Their study demonstrated that 3D imaging has several clinical advantages. Specifically, the 3D group experienced a significantly lower rate of global ischemia (45.8% vs. 69.7%; p = 0.03) and a higher enucleation rate (62.5% vs. 37.5%; p = 0.02) than the US group. Additionally, the use of 3D reconstructed images resulted in a markedly lower incidence of collecting-system violations (10.4% vs. 45.5%; p = 0.003). These findings suggest that 3D imaging provides superior anatomical visualization, contributing to a reduced ischemic time and improved surgical precision [19]. Amparore et al. reported that the application of 3D reconstructed images during RAPN resulted in a significantly smaller decline in renal function compared to cases where 3D imaging was not utilized (− 10% vs. − 19.6%; p = 0.002). It should be noted that the majority of these trials involved only a few cases with varied background factors, which limits the strength of the scientific evidence.

In a systematic review by Piramide et al., 3D guidance for NSS was associated with a significant reduction in the transfusion rate (1.3% vs. 7.2%, p < 0.01), with no significant differences observed in the rates of conversion to radical nephrectomy, minor and major complications, changes in the glomerular filtration rate, or surgical margins [18]. However, most studies included in this review were retrospective and reported preliminary experiences, which does not provide sufficient evidence.

Recent studies using PSM have reported that the use of 3D reconstructed images improves surgical outcomes. Kobayashi et al. demonstrated that cases utilizing a system that overlays 3D models onto endoscopic images exhibited a higher proportion of preserved renal parenchyma volume and a tendency toward better postoperative renal function compared with cases that did not use this system [20]. Additionally, a relatively large multicenter PSM analysis conducted by Michiels et al. found that 3D image guidance was associated with a significantly lower rate of major postoperative complications (3.8% vs. 9.5%, p = 0.04), a less pronounced decline in eGFR (−5.6% vs. −10.5%, p = 0.002), and a higher achievement rate of trifecta (55.7% vs. 45.1%, p = 0.005) [21].

Our results also demonstrated that the group using 3D navigation had a significantly lower rate of postoperative complications and an improved trifecta achievement rate, supporting their findings. Interestingly, in the initial cohort, the 3D-R RAPN group showed longer operative time and a higher rate of complications compared with the control group, whereas these outcomes were significantly improved after PSM. This apparent discrepancy can be explained by differences in patient backgrounds and case complexity over time. In Japan, the indication for RAPN has expanded in recent years following its inclusion in the national insurance coverage system. Initially, robotic partial nephrectomy was reimbursed only for limited indications, but subsequent revisions of the reimbursement criteria allowed its use in larger and more complex renal tumors. As a result, the number of high-complexity RAPN cases has increased substantially in recent years. Because our hospital serves as a tertiary referral center covering a wide region, more complex cases have tended to accumulate in recent years, when most 3D-RAPN procedures were performed. Consequently, the 3D-R RAPN group included a greater proportion of patients with larger tumors, higher R.E.N.A.L. scores, and anatomically challenging features, resulting in longer operative times and higher complication rates in the unmatched analysis. After adjusting for these baseline differences by PSM, the outcomes became more comparable between groups, and the 3D-R RAPN group demonstrated a higher trifecta achievement rate. These findings suggest that intraoperative 3D navigation may contribute to improved surgical outcomes in RAPN.

In this study, although WIT was significantly shorter in the 3D navigation group, the reduction was only 2.5 min and had little impact on the total operative time. However, the approximately 12% decrease in WIT and the lower rate of postoperative complications may indicate that intraoperative 3D navigation enhanced the surgeon’s anatomical understanding, allowing more accurate recognition of renal structures during tumor excision and more precise renal reconstruction, which led to higher-quality surgery.

The use of 3D reconstructed imaging in preoperative planning has been shown in several studies to assist in deciding between partial and radical nephrectomies [22]. Additionally, some reports suggest that using head-mounted displays with virtual reality could further support this decision-making process [23].

Several 3D imaging reconstruction software programs can identify the perfusion areas of the renal artery branches, thereby facilitating the selection of appropriate clamping techniques [24–26]. These 3D reconstructed images are utilized based on the Voronoi diagrams to provide detailed information about perfusion areas, allowing for accurate predictions of the perfusion territories [27, 28]. It has also been reported that selective renal artery clamping guided by these 3D reconstructed images contributes to the preservation of renal function [29]. The REVORAS system also calculates perfusion areas based on Voronoi diagrams, enabling an accurate reconstruction of these regions and facilitating selective clamping.

The requirement for specialized knowledge and significant time investment have been notable drawbacks in the use of 3D reconstruction images. Porpiglia’s report highlighted that creating 3D models requires 4–6 h of work by bioengineers, with their constant presence needed during surgical procedures [19].

Fortunately, recent advances in technology and artificial intelligence have significantly improved this process. With the REVORAS system, high-quality and precise 3D images can be generated automatically without depending on the specialized support of radiologic technologists or engineers.

In the present study, the main objective was to evaluate the impact of intraoperative 3D navigation on surgical outcomes; therefore, a detailed quantitative analysis of modeling efficiency or cost was not included. Based on our institutional experience, however, the modeling process generally required about 5–10 min per case, and 3D reconstruction was successfully achieved in all cases. Major anatomical structures—including the renal parenchyma, arteries, veins, and urinary tract—were automatically extracted in 90% cases, although a few required minor manual refinements, such as adjustments of peripheral renal arteries. Compared with previous reports, this high level of automation and short processing time seem to reduce the time and personnel burden associated with the clinical application of 3D navigation. A prospective study systematically evaluating modeling efficiency and cost is currently underway at our institution, and the results will be presented in future work.

Although these technological advances have lowered the barriers to clinical application, full automation has not yet been achieved, and further refinements are expected.

This study had several limitations. First, it was conducted retrospectively using PSM rather than a randomized design that could have eliminated selection bias. Second, although we restricted the analysis to experienced surgeons to minimize differences in baseline characteristics and reduce learning curve bias, variability in surgical experience still existed within this group. Third, due to the structure of this study, the two groups were operated on during different time periods, and therefore the potential influence of a time-period bias cannot be completely excluded. Finally, this was a single-institution study with a relatively small sample size.

Further large-scale prospective studies are warranted to validate our findings.

## Conclusion

This study is among the first to explore the efficacy of the newly developed 3D reconstruction application. This application allows the automatic extraction of 3D reconstructed images for all organs, with semi-automated extraction of tumors. The use of 3D technology appears to have the potential to improve surgical objectives, such as reducing warm ischemia time, decreasing perioperative complication rates, and increasing trifecta achievement rates, which may contribute to enhancing the overall quality of surgical procedures.

## Supplementary Information

Below is the link to the electronic supplementary material.

Supplementary file1 (MP4 158258 KB)

Supplementary file2 (MP4 158258 KB)

## Abbreviations

3D: Three-dimensional
CT: Computed tomography
EBL: Estimated blood loss
eGFR: Estimated glomerular filtration rate
OT: Operative time
PSM: Propensity score matching
RAPN: Robot-assisted partial nephrectomy
RCC: Renal cell carcinoma
RT: Robotic surgery time
US: Ultrasound
WIT: Warm ischemia time
